# *Helicobacter pylori* infection process: from the molecular world to clinical treatment

**DOI:** 10.3389/fmicb.2025.1541140

**Published:** 2025-02-27

**Authors:** Meijing Yi, Silan Chen, Xinying Yi, Fan Zhang, Xuan Zhou, Meiyan Zeng, Houpan Song

**Affiliations:** ^1^School of Traditional Chinese Medicine, Hunan University of Chinese Medicine, Changsha, China; ^2^Hunan Provincial Key Laboratory of Traditional Chinese Medicine Diagnostics, Hunan University of Chinese Medicine, Changsha, China

**Keywords:** *Helicobacter pylori*, pathogenic mechanism, treatment, antibiotic resistance, probiotics

## Abstract

*Helicobacter pylori* is a gram-negative microaerophilic microorganism intricately associated with chronic gastrointestinal disorders and gastric cancer. *H. pylori* can cause various upper digestive tract diseases, including chronic gastritis, peptic ulcer, gastroesophageal reflux disease, and gastric cancer. The bacterium exhibits a variety of pathogenic mechanisms, including colonization, the expression of virulence factors, and the development of drug resistance. This article presents a comprehensive review of *H. pylori* pathogenesis, emphasizing recent research advancements concerning the cytotoxin-associated gene A, vacuolating cytotoxin, outer membrane proteins, and other virulence factors. Additionally, it examines the molecular mechanisms underlying drug resistance and evaluates the efficacy of conventional therapeutic approaches. Recently, researchers have attempted novel therapeutic regimens, including probiotics and Chinese medicine-assisted therapies, to enhance therapeutic effects. This article aimed to offer an overview of the academic community’s comprehension of *H. pylori* infection and to highlight the current treatment options.

## 1 Introduction

*Helicobacter pylori* is a gram-negative, transmissible bacterium within the *Helicobacteriaceae* family (Li et al., 2023). Typically, *H. pylori* exhibits a spiral morphology, although it can also assume a rod-like form. Additionally, the organism may adopt a spherical shape following extended *in vitro* culture or exposure to antibiotic treatment. *Helicobacter* species are categorized into two types based on their ecological niche: gastric and enteric. Among the *Helicobacter* species adapted to humans, *H. pylori* is classified as a gastric species (Nabavi-Rad et al., 2022). The infection rate of *H. pylori* exceeds 50% worldwide and is even higher in developing countries where sanitation and hygiene practices may be inadequate (Guo et al., 2024).

Since the discovery of *H. pylori* in 1983, continuous research has been conducted to eradicate it for decades (Hooi et al., 2017). *H. pylori* is becoming increasingly resistant to antibiotic treatments, complicating the management of its infections in the stomach. These infections result in significant changes to the composition of the gastrointestinal microbiota (Sharndama and Mba, 2022). The two main aspects related to the pathogenic basis of *H. pylori* are as follows. The cytotoxin-associated gene pathogenicity island (Cag PAI) is a strain-specific *H. pylori* component. This gene locus encodes the type IV secretion system (T4SS) island terminal gene product, cytotoxin-associated protein gene A (CagA) (Jang et al., 2022). Another *H. pylori* locus associated with an increased risk of gastric cancer is the vacuolating cytotoxin (VacA), which encodes a secreted toxin. *In vitro*, the VacA protein induces intracellular vacuole formation, cytotoxicity, and cell apoptosis. Outer membrane proteins (OMPs) are widely believed to be implicated in the pathogenic process of *H. pylori* by facilitating the *in vivo* attachment of *H. pylori* to gastric epithelial cells (Chmiela et al., 2018). *H. pylori* can lead to the formation of biofilms, thereby increasing drug resistance (Sharndama and Mba, 2022).

During chronic infection, *H. pylori* is localized in the mucosal layer of the stomach, particularly inside the deeper part of the invagination (the glands), and can persist throughout the host’s life (Sharndama and Mba, 2022). While other microorganisms are unable to survive in the harsh acidic environment (pH <2) of the stomach, *H. pylori* can colonize the gastric mucosal layer, reaching the epithelial cell layer (pH 5–6) (Algood and Cover, 2006). Nevertheless, curing *H. pylori* infection presents significant challenges, and with the pervasive use of therapeutic drugs, coupled with the specific adaptations of *H. pylori*, antibiotic resistance in *H. pylori* is on the rise. A comprehensive understanding of the mechanisms underlying *H. pylori* drug resistance is required to resolve this issue. In this research, we reviewed the colonization process of *H. pylori*, the function of secreted virulence factors, and the mechanisms underlying the development of resistance.

## 2 Mechanisms of persistent colonization of *Helicobacter pylori*

The colonization of *H. pylori* and the establishment of disease and infection rely on four main stages: initial adaptation to the ultra-acidic environment of the gastric mucosa, gradual movement and penetration into the epithelial cell barrier, binding to specific receptors, and ultimately, the production of a series of tissue damage and other deleterious effects (Sharndama and Mba, 2022). *H. pylori* infections are transmitted between hosts through fecal-oral or oral-oral routes (Alipour, 2021). The stomach’s extremely acidic pH (1–2) in the human gastric lumen is the most critical antimicrobial property, killing most bacteria and preventing their proliferation in the lumen (Salama et al., 2013).

### 2.1 Basic gastric conditions

*H. pylori* mostly enters the stomach through the digestive tract. The stomach produces gastric acid in its physiological state, forming an intragastric pH of 1–2. Gastric acid kills bacteria, and the stomach undergoes peristalsis and evacuation, severely restricting intestinal colonization (Hunt et al., 2015). After entering the stomach, *H. pylori* survives in the gastric lumen for only a few minutes, necessitating rapid transfer to the gastric epithelial cell surface, which is filled with a mucus layer serving as a barrier. The mucus layer in the stomach forms a physical barrier to bacterial penetration and may serve as a scaffold for binding host antimicrobial compounds (Salama et al., 2013).

### 2.2 Escape from the gastric liquids

**Figure 1:**
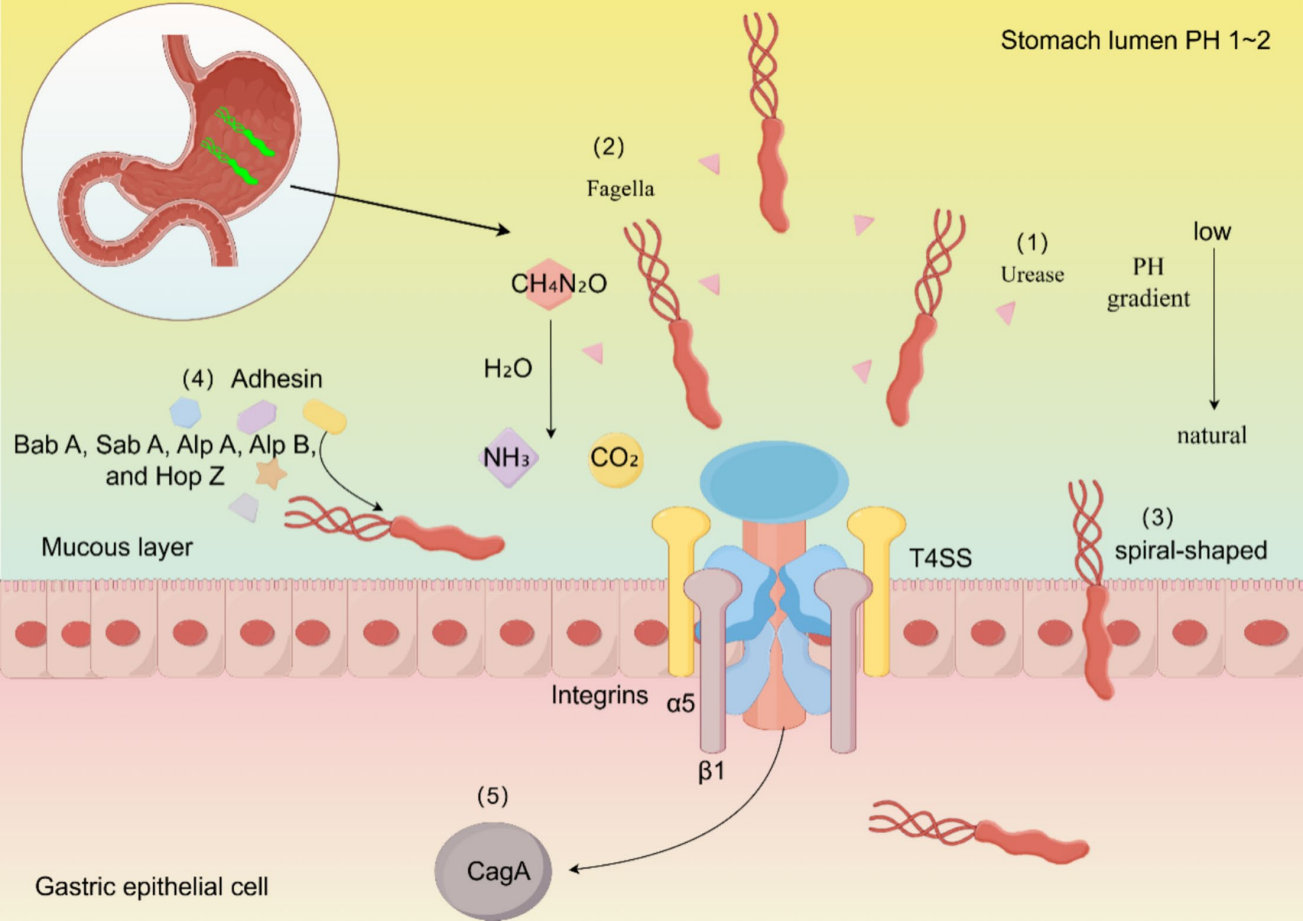
Mechanisms of persistent *H. pylori* colonization. The colonization process of *H. pylori* is primarily completed through the following aspects: (1) *H. pylori* increases stomach pH by producing ammonia via urease. (2) *H. pylori* exhibits motor chemotaxis through its flagellum. (3) *H. pylori* enters host cells more effectively in its spiral form. (4) SabA, BabA, and other various expressions of adhesins help *H. pylori* adhere to host cells. (5) CagA affects the metabolism of host cells and disrupts their polarity, thereby enhancing the colonization of *H. pylori*.

How does *H. pylori* escape from the particularly dangerous gastric liquids? First, *H. pylori* possesses a distinctive spiral-shaped body with special flagella at one end, which facilitates its flexible motility, and it contains a highly active urease enzyme. *H. pylori* employs two main weapons to penetrate the mucosal layer: flagellum and urease (Figure 1). *H. pylori* produces two types of ureases: one is located in the bacterial cytoplasm, while the other is found on the surface. The external is primarily produced during the lysis of other bacterial cells and functions at a pH of 5.0–8.0, while the internal function is at a pH of 2.5–6.5. Urease degrades urea to generate ammonium ions, neutralizes gastric acid, and enables *H. pylori* to temporarily survive in the highly acidic gastric lumen, thereby protecting *H. pylori*. Urease also modifies the elasticity of gastric mucin to facilitate flagella, while the helical form of flagellar movement assists *H. pylori* in crossing the mucosal layer. Typically, gastric mucin forms a gel at low pH that effectively traps bacteria. However, the urease-catalyzed production of ammonium ions increases the pH to near neutrality, and the mucus gel is transformed into a viscoelastic solution through which *H. pylori* can swim and enter the host cell. Spiral-like cell shapes of *H. pylori* are believed to facilitate its penetration into the host cell by a helical mechanism. Mutations in flagellin genes, including *fliD*, *flaA*, and *flaB*, that alter the spiral shape of flagella can hinder the colonization of *H. pylori* in the gastric mucosa (Sharndama and Mba, 2022). The flagella of *H. pylori* are capable of both motility and adhesion. In the presence of relatively high acidity, which is increased in the gastrointestinal tract, the flagellum functions as a proton-motive force. At this pH, the flagellum tends to swim faster, powering protein movement (Salama et al., 2013).

In addition to the enzymes associated with colonization, *H. pylori* possesses carbonic anhydrase, which is crucial for maintaining a stable acid–base balance both intracellularly and extracellularly. *H. pylori* encodes two distinct carbonic anhydrases, which are critical for its survival in the acidic environment of the stomach (Bury-Moné et al., 2008; Maresca et al., 2013). Gastric acid is produced by parietal cells, and its secretion depends on the hydrogen-potassium adenosine-triphosphatase (H^+^/K^+^-ATPase). *H. pylori* inhibits the activity of this enzyme through its fatty acids and suppresses the expression of the α-subunit gene via the CagL protein. *H. pylori* can downregulate the mRNA expression of H^+^/K^+^-ATPase, thereby diminishing its synthesis, reducing gastric acid secretion, and consequently promoting its survival in the stomach (Beil et al., 1994; Osawa et al., 2006; Saha et al., 2010).

### 2.3 Other colonization mechanisms

During antibiotic treatment or prolonged *in vitro* culture, *H. pylori* may change into a spherical shape for better movement *in vivo*. Under adverse environmental conditions such as low nutrients, dryness, lack of oxygen, and exposure to antibiotics, *H. pylori* can survive by changing from a spiral to a coccoid form (Sharndama and Mba, 2022). Furthermore, *H. pylori* utilizes L-lactate to promote complement resistance and successfully initiate gastric colonization. Significantly lower levels of C3 complement content were observed in the gastric juices of *H. pylori*-positive individuals compared to *H. pylori*-negative individuals. Complement restricts intragastric colonization by *H. pylori*, particularly in the gastric body. Complement mediates direct cytolysis and promotes local immune responses. Previous studies have shown that lactic acid in the blood can help *H. pylori* evade conventional attacks by the body’s immune system (Hu and Ottemann, 2023). Biofilm formation has recently been acknowledged as contributing to colonization. Biofilms are surface-associated bacterial communities embedded in a hydrated matrix of extracellular polymers. *H. pylori* can form varying degrees of biofilms *in vitro*, and the formation intensity closely correlates with the strain’s drug resistance and acid resistance (Lin et al., 2024).

## 3 Virulence factor

*H. pylori* is a highly heterogeneous bacterium whose virulence varies geographically (Yamaoka, 2010). *H. pylori* enhances its pathogenicity by secreting several toxins, including CagA, VacA, and outer membrane inflammatory protein (OipA).

### 3.1 CagA

CagA is a well-studied virulence factor of *H. pylori*. To date, CagA is the sole protein identified as being secreted by Cag T4SS (Cover et al., 2020). *H. pylori* strains contain a 40 kb region of chromosomal DNA known as the Cag PAI. The product of the Cag PAI, CagA, is translocated to the gastric epithelium and induces cellular signaling. The T4SS, which is encoded by the Cag PAI, contains nearly 31 genes (Sharndama and Mba, 2022). Upon contact with the host epithelium, T4SS possesses a syringe-like structure that transports effector proteins CagA and peptidoglycan (PG) into the epithelium (Ansari and Yamaoka, 2020).

CagA is a protein (128–145 kDa) containing a structured N-terminal region and a C-terminal tail (Wang et al., 2023). It is clinically distinguished into two types: CagA-positive and CagA-negative strains (Takahashi-Kanemitsu et al., 2020). CagA is recognized by human serum antibodies and gastric mucosal IgA antibodies (Smirnova et al., 2022). The N-terminal consists of three different domains (domains I–III) to form a new protein structure. Each repeat region of the CagA protein contains a Glu-Pro-Ile-Tyr-Ala (EPIYA) motif, including a tyrosine phosphorylation site. EPIYA motifs are classified into four types based on their respective conserved flanking sequences: EPIYA-A, -B, -C, and -D (Ansari and Yamaoka, 2020). Multiple studies have indicated that tyrosine phosphorylation sites in the EPIYA motif of CagA directly influence *H. pylori* pathogenicity (Wang et al., 2023). Compared with Cag PAI-negative *H. pylori* strains, Cag PAI-positive strains stimulate gastric epithelial cells to produce high levels of pro-inflammatory cytokines, including interleukin (IL)-8, which is partly associated with the expression of CagL (Cover et al., 2020; Tegtmeyer et al., 2017).

CagA is produced in the bacterial cytoplasm and enters the host cell across the bacterial biofilm and host cell membranes using the Cag T4SS. The T4SS of *H. pylori* forms needle-like hairs that bind to polar-regulated kinase 1b (PAR1b) to form the CagA-PAR1b complex, which induces host cell attachment and polarity defects and assists in the entry of CagA molecules into host cells (Ansari and Yamaoka, 2020; Wang et al., 2023). Once CagA reaches the host cell cytoplasm, tyrosines in the EPIYA motif are phosphorylated by the host SRC and ABL family kinases (Salama et al., 2013). Tyrosine-phosphorylated CagA interacts with many intracellular proteins, leading to modifications in protein function (Takahashi-Kanemitsu et al., 2020). Upon entering the cytoplasm through the T4SS, CagA alters both phosphorylation-dependent and phosphorylation-independent host cell signaling. As a result of phosphorylation, CagA binds to the phosphatase SHP-2 to affect cell adhesion, spreading, and migration (Kao et al., 2016).

At least 17 genes in *H. pylori* Cag PAI are required for the translocation of CagA into the host cell. T4SSs are large membrane-associated transport complexes (enormous mushrooms) in gram-negative and gram-positive bacteria, along with some archaea which transport several substrates, including proteins and DNA (Cover et al., 2020). They function as a contact-dependent secretion system comprising a bacteriophage and multiple ATPases and usually comprise 11 VirB proteins (encoded by the *VirB*1–11 gene) and a coupling protein (VirD4, an ATPase) (Tran et al., 2024). The two primary functions of T4SSs are the horizontal transfer of DNA between bacteria and the transportation of effector proteins to target cells (Cabezón et al., 2015). T4SSs encompass the outer membrane core complex (OMCC) and inner membrane complex (IMC). The OMCC is approximately 41 nm in diameter and comprises five major components (CagY, CagX, CagT, Cag3, and CagM). The IMC fraction has 6-fold symmetry and comprises three concentric rings around the central channel with three ATPases (VirB4, VirB11, and VirD4), VirB3, and VirB8 (Macé et al., 2022).

One mechanism involving host cells and T4SS is the interaction between Cag proteins and host cells. Integrins are transmembrane cell adhesion molecules that mediate cell–cell and cell–matrix interactions, anchoring cells to the underlying matrix. Aggregated integrins assemble into actin-rich structures known as adhesion patches. Once T4SS hairs are formed, *H. pylori* deliver PG, chromosomal DNA, heptose 1,7-bisphosphate, and CagA effector proteins, which then phosphorylate EPIYA motifs through oncogenic Src and Abl tyrosine kinases. To date, there is no evidence that the Cag T4SS element is directly inserted into the host cell (Tran et al., 2024).

*H. pylori* mediates attachment to target cells by producing extracellular filamentous structures called F pili in response to contact with gastric epithelial cells (Costa et al., 2021). In polarized epithelial cells, integrins are normally expressed on the basolateral surface and protected by tight junctions (TJs) and E-cadherin-based adhesion junctions (AJs). *H. pylori* can secrete the serine protease high-temperature requirement protein, serine proteases, and chaperonins. This protease effectively disrupts TJs and AJs, allowing bacteria to enter the cellular monolayer hours after infection. Fungal hair formation and T4SS activation occur primarily at the basolateral site. This enables bacteria to traverse polarized epithelial cells and reach integrins to inject CagA into the basolateral membrane (Tegtmeyer et al., 2017).

### 3.2 VacA

VacA is the second-most extensively studied *H. pylori* virulence factor. Soon after the discovery of *H. pylori*, it was reported that a protein in *H. pylori* broth culture filtrates can cause the formation of large intracellular vacuoles in cultured mammalian cells. The *H. pylori* protein responsible for this effect is encoded by the chromosomal gene *vacA* (Cover and Blanke, 2005). The difference in vacuolating abilities is determined by variations in the three regions of the *vacA* gene: the signal (s1 and s2), intermediate (i1 and i2), and middle regions (m1 and m2) (Chen et al., 2018).

**Figure 2:**
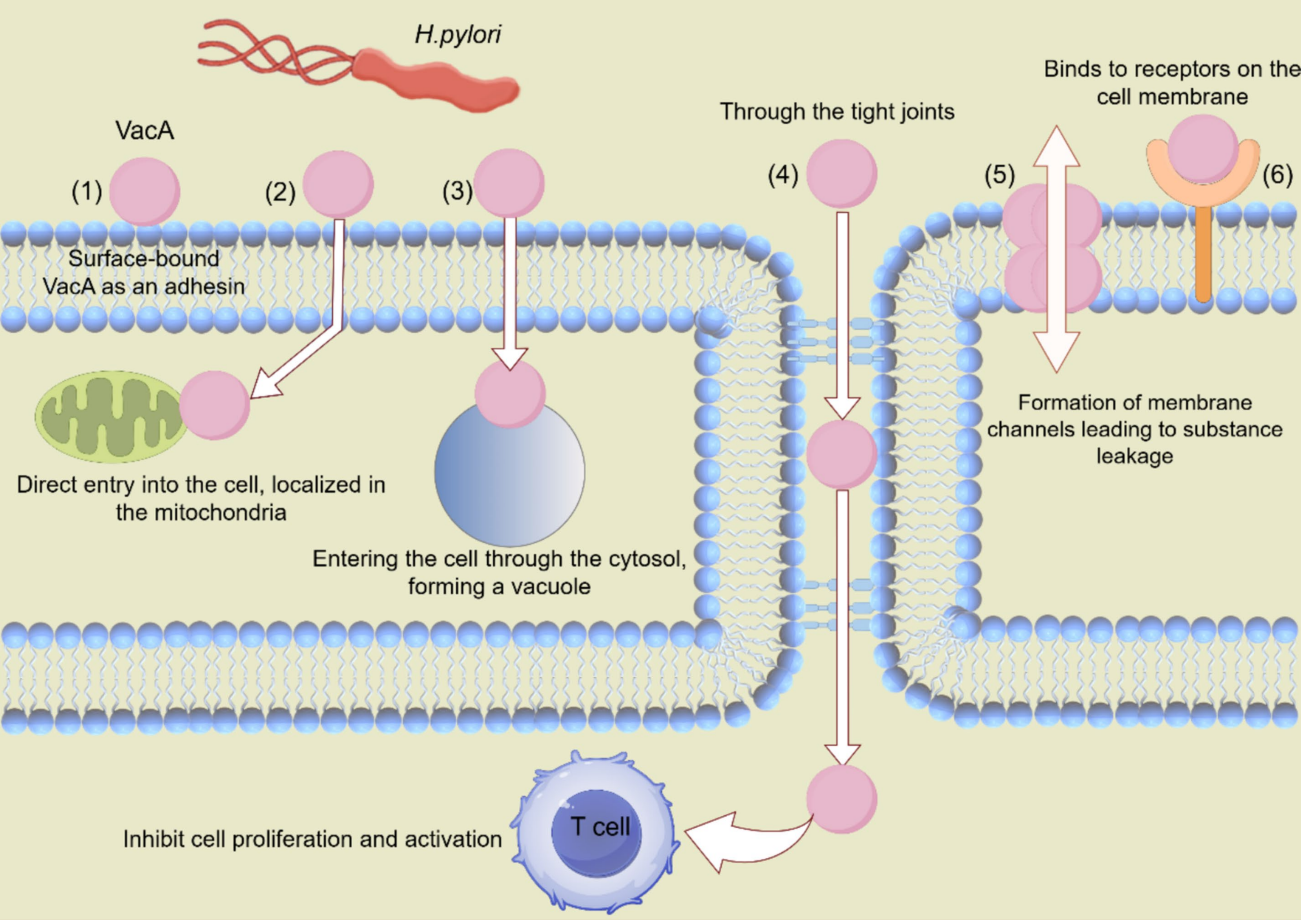
Various mechanisms through which VacA contributes to *H. pylori* colonization. VacA proteins affect cellular processes in various ways, including the following: (1) VacA can be surface-bound and act as an adhesion factor. (2) VacA can be directly ingested by cells and transported to mitochondria to induce apoptosis. (3) VacA can be ingested, inducing cavitation. (4) VacA inhibits T cell activation and proliferation through tight junctions. (5) VacA is composed of membrane channels that cause nutrients to leak into the extracellular space. (6) VacA binds to cell membrane receptors and initiates pro-inflammatory reactions.

All strains of *H. pylori* isolated from humans contain the *vacA* gene, suggesting that VacA production is important for *H. pylori* colonization or persistence in the stomach (Cover and Blanke, 2005). Approximately 50% of *H. pylori* strains secrete VacA. VacA protein is produced as a 140 kDa prototoxin and is cleaved to a mature form of 95 kDa upon secretion (Kusters et al., 2006). The effects of VacA include adhesion to cell membrane surfaces, formation of membrane channels leading to nutrient leakage, disruption of endosomal and lysosomal activities, effects on integrin receptor-induced cell signaling, induction of apoptosis, and immunomodulatory inhibition of T-cell activation and multiplication (Cover and Blanke, 2005; Kusters et al., 2006). VacA forms pores in epithelial cell membranes, inducing the release of urea and anions from host cells. Vacuole formation on the host cell’s cytoplasmic membrane renders the host cell vulnerable to urease activity (Sharndama and Mba, 2022). It also increases the transcellular permeability, leading to the release of nutrients and cations (Kusters et al., 2006). Some toxins enter the cell through the pores, while others reside outside. These toxins bind to specific receptors that mediate their entry. The toxins undergo a series of reactions once they enter the cell (Figure 2).

#### 3.2.1 Effect of VacA on lysosomes

A crucial component of the endolysosomal system is the transient receptor potential membrane channel mucolipin 1 (TRPML1), an endolysosomal calcium channel. VacA targets TRPML1 to destroy lysosomal transport. Lysosomes and autophagy are safeguards that maintain the stability of the endo-environment, function to remove invading pathogens, and prevent malignant transformation. Elevated intracellular calcium levels in VacA^+^-treated cells strongly suggest impaired TRPML1 activity. These results confirm that VacA disrupts TRPML1 activity and causes disruption of lysosomal calcium homeostasis. Both TRPML1-deficient and VacA-intoxicated cells exhibit dysfunctional endolysosomes and autophagic vesicles. A small molecule TRPML1 agonist can reverse VacA harmful impact on endolysosomal trafficking, leading to the elimination of intracellular bacteria. This indicates TRPML1 could be a target for treating chronic *H. pylori* infections (Capurro et al., 2019).

#### 3.2.2 Induction of cellular vacuolization

VacA induces an acidic environment within vesicles, and the vesicles have endocytosis activity (Papini et al., 1994). Current models of VacA-induced vacuolization suggest that VacA is internalized by the cell and forms membrane channels in the membranes of late endocytic compartments. One possibility is that VacA-induced vesicles may result from the fusion of multiple smaller endosomal compartments. Another possibility is that vesicles may form from late endosomes without the need to fuse different compartments (Cover and Blanke, 2005).

#### 3.2.3 Effects on mitochondria

Although many VacA-mediated effects arise directly or indirectly from membrane binding and pore formation, VacA also enters the cytosol and accumulates in the inner mitochondrial membrane, activating endogenous mitochondrial channels, thereby inducing apoptosis (Kusters et al., 2006). VacA also induces mitochondrial alterations. p34, the N-terminal fragment of VacA, targets mitochondria when expressed in the cytoplasm and induces the release of cytochrome c, leading to a decrease in mitochondrial transmembrane potential, which coincides with alterations in mitochondrial membrane permeability. VacA-induced modifications of mitochondria have been reported to decrease the concentration of ATP in cells and impair cell cycle progression (Cover and Blanke, 2005; Galmiche et al., 2000; Willhite and Blanke, 2004).

#### 3.2.4 Effects on signaling pathways

Two classes of mitogen-activated protein kinases (MAPK) and activated transcription factor 2 (ATF2) signaling pathways were activated by the addition of VacA to the human gastric adenocarcinoma cell line (AZ-521) within 10 min. The inhibition of p38 kinase activity (SB203580) did not block VacA-induced vacuolization or VacA-induced cytochrome c release, indicating that VacA-induced activation of the p38/ATF2 signaling pathway is independent of its effects on late endocytosis of compartments and mitochondria (Nakayama et al., 2004).

#### 3.2.5 Effects on T-lymphocytes

VacA secreted by *H. pylori* appears to penetrate deeper into tissues where it interacts with other associated cell types, including granulocytes, monocytes, B cells, and T cells. IL-2 is necessary for the proliferation and survival of T cells (Kusters et al., 2006). VacA inhibits IL-2 production and down-regulates the surface expression of IL-2 receptor α. VacA effectively blocks T cell proliferation by inducing cellular G1/S phase cycle blockade. VacA inhibits the nuclear translocation of the nuclear factor of activated T cells by blocking the influx of calcium from the extracellular environment, resulting in the inability of T cells to express and secrete IL-2 (Gebert et al., 2003).

#### 3.2.6 Pro-inflammatory effects of VacA

VacA has been reported to be a mast cell chemokine that promotes the production of pro-inflammatory factors, tumor necrosis factor-alpha (TNF-α), macrophage inflammatory protein-1, IL-1, IL-6, IL-10, and IL-13 by mast cells (Supajatura et al., 2002). Moreover, VacA activates p38 in macrophages and neutrophils, increasing COX-2 expression and promoting inflammatory expression by producing pro-inflammatory prostaglandins (Boncristiano et al., 2003).

### 3.3 Outer membrane proteins

Approximately 4% of the *H. pylori* genome is predicted to encode OMPs, some of which may function as adhesins (Yamaoka, 2010). At least 32 *H. pylori* OMPs have been identified, most of which are associated with bacterial adhesion (Yamaoka et al., 2006). The OMPs of *H. pylori*, such as blood group antigen-binding adhesin (BabA) and salivary acid-binding adhesin (SabA), play a significant role in enhancing the bacterium’s adhesion affinity to host blood group antigens. Specifically, BabA can bind to ABO/Lewis b (Leb) blood group antigens (Yamaoka et al., 2006), while SabA prefers to bind to sialylated glycans (sialyl-Lewis X, sLex) (Mahdavi et al., 2002). These proteins help the bacteria colonize the gastric mucosa and initiate infection by binding to specific glycan antigens on the surface of host cells. The high-affinity binding of these adhesins allows *H. pylori* to firmly attach to the gastric mucosal surface, thereby evading clearance by the host immune system.

### 3.4 OipA

In 2000, the HP0638 gene was named OipA. *H. pylori* OipA is a crucial virulence factor that is associated with increased IL-8 secretion and inflammation, and it is an important clinical manifestation of peptic ulcer (Yamaoka et al., 2000). OipA expression is regulated by slip-strand mismatches determined by the number of Ct dinucleotide repeats in the 5′-region of the *oipA* gene (Yamaoka, 2010). Strains containing Cag PAI usually also have OipA, with a functional state of “on” (Yamaoka et al., 2002). Both OipA and Cag PAI are required for full activation of the IL-8 promoter but act through different pathways, and upstream of IRF-1, only OipA is involved in the STAT1-IRF1-ISRE pathway (Yamaoka et al., 2004). OipA is required for the nuclear localization of β-catenin, suggesting that different *H. pylori* components can induce the nuclear translocation of β-catenin and increase the homeostatic levels of free β-catenin, leading to the opening of intercellular junctions and proliferation (Franco et al., 2008).

### 3.5 SabA

SabA is encoded by JHP662 (HP0725). This gene encodes a 651 amino acid protein (70 kDa) that belongs to the large-hop family of *H. pylori* outer membrane protein genes, including *babA*. *H. pylori* BabA adhesin binds Leb antigen on glycoproteins, while SabA adhesin binds sLex antigen on membrane glycolipids. *H. pylori* adheres firmly to the stomach by binding its surface adhesin BabA to the Leb antigen on the surface of the host’s gastric mucosal epithelial cells. Accordingly, *H. pylori* adhesion during chronic infection may involve two separate receptor-ligand interactions, one mediated by Leb and the other by weaker sLex-mediated adhesion. sLex-mediated adhesion, as well as its metastable switching mechanism, may benefit *H. pylori* by allowing it to evade sites where host defenses are most reactive (Mahdavi et al., 2002). SabA has been suggested to act as a selectin mimetic by binding to sialyl-(di)-Le^x/a^ sphingolipids and promoting membrane adhesion and attachment. At sites of active local inflammatory response, it is possible that *H. pylori* undergoes a phase shift and shuts down the sLex-binding capacity, allowing nonfunctional SabA to avoid contact with sialylated lymphocytes or other defense cells in close contact (Yamaoka et al., 2006).

### 3.6 Outer membrane vesicles

**Figure 3:**
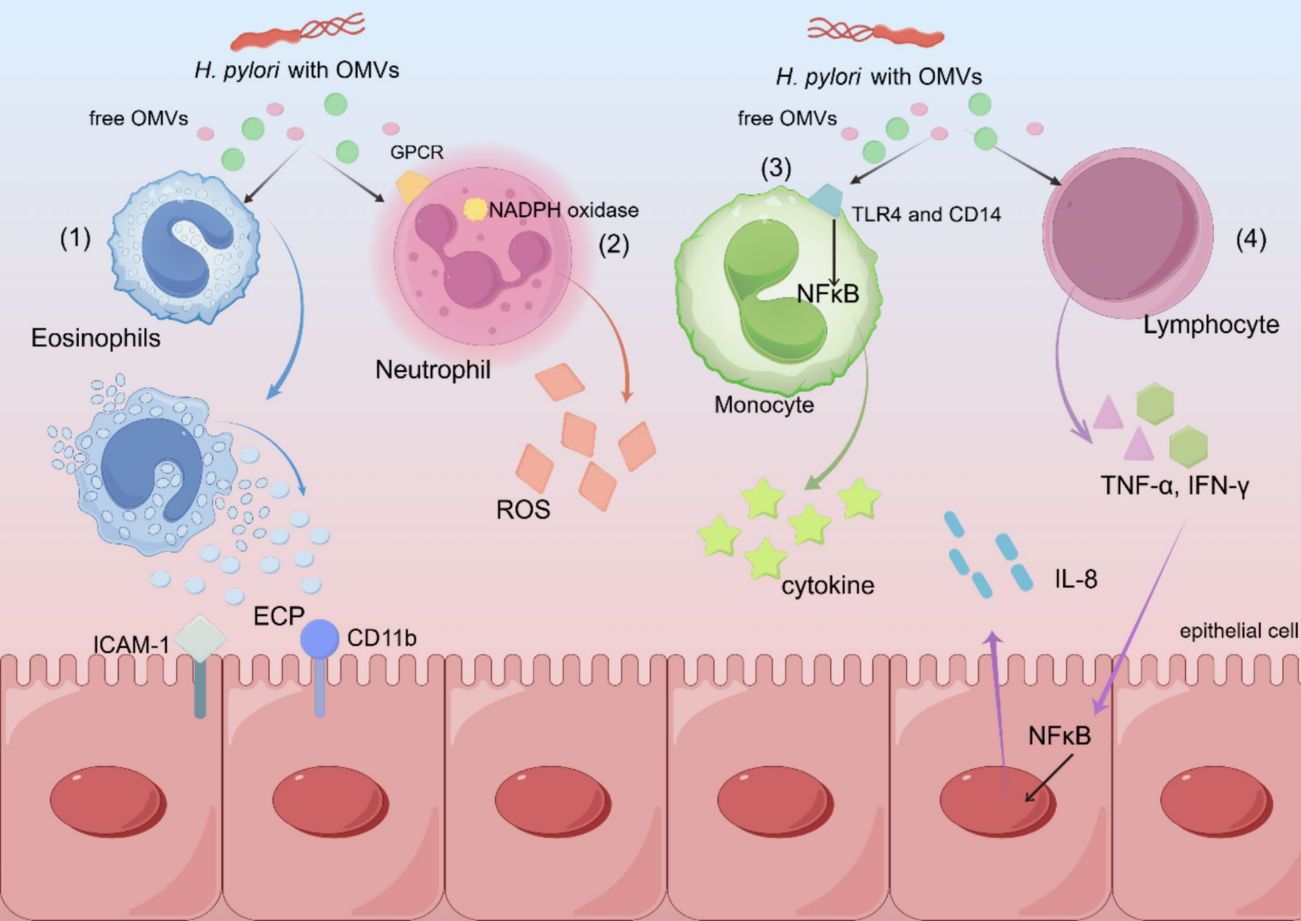
Schematic diagram of various mechanisms by which *H. pylori* OMVs exert pathogenic effects. (1) *H. pylori* OMVs induce the expression of ICAM-1 and CD11b, resulting in degranulation and the release of ECP. (2) Neutrophils are activated by a G-protein pathway involving G-protein coupled receptors and kinases, which leads to the activation of NADPH oxidase, resulting in an oxidative burst and the production of ROS. (3) The binding of TLR4 and CD14 to OMVs leads to the activation of mononuclear NF-κB and cytokine release. (4) Increased T cell activation and secretion of cytokines, including TNF-α and IFN-γ, stimulate the gastric epithelium to release the pro-inflammatory cytokine IL-8.

Gram-negative bacteria commonly shed vesicles, known as OMVs, during normal growth. OMVs are spherical, double-membrane vesicles (approximately 20–350 nm) that are commonly expressed on the surface of *H. pylori* and other gram-negative bacteria. OMVs may contain inner and outer membrane proteins, periplasmic proteins (Turner et al., 2018), lipopolysaccharides (LPS) (Kaparakis et al., 2010), PG, DNA, and toxins (Kesty et al., 2004). *H. pylori* OMVs of different sizes enter epithelial cells by giant cytosolic drinking, lattice protein, and niche protein-dependent endocytosis. Inhibition of niche proteins results in the greatest reduction in the entrance of small OMVs into host cells. Conversely, the entry of larger OMVs into epithelial cells is inhibited by all endocytosis mechanisms. Proteins associated with *H. pylori* survival or virulence are common in large and small OMVs, including urease A and B subunits, neutrophil-activating protein VacA, and pore protein HopA (Figure 3). Unlike smaller proteins, larger proteins contain several adhesins, including SabA, BabA, iron-modulating proteins, the Hop family of outer-membrane proteins, and many flagellar basal and hook proteins (Turner et al., 2018).

VacA potentially facilitates the uptake of OMVs, and once OMVs bind to gastric epithelial cells, their components undergo endocytosis and are transported in endosomes, which fuse with lysosomes and further impair cellular function (Parker et al., 2010). In response to vesicle-associated virulence factors that induce gastric and immunoinflammatory cascades, *H. pylori* OMVs fractions stimulate pro-inflammatory and anti-inflammatory cytokines, respectively. Excessive eosinophil degranulation causes epithelial damage by releasing cytotoxic granule proteins, including eosinophil cationic protein (ECP). *H. pylori* OMVs induced degranulation of human eosinophils, releasing ECP by inducing enhanced expression of epithelial intracellular adhesion molecule-1 (ICAM-1) and CD11b integrin on the eosinophil surface (Ko et al., 2015).

OMVs delivered *H. pylori* virulence factors can target host transcriptional regulators, including nuclear factor-κB (NF-κB), mitogen-activated protein kinase, and extracellular signal-regulated kinase. Consequently, OMVs regulate the expression of genes involved in the inflammatory response, including the secretion of IL-8, TNF-α, or IL-1β, along with their proliferative and oncogenic effects (Chmiela et al., 2018; Maeda et al., 2001).

## 4 Evasion of innate immune recognition

*H. pylori* colonization in the stomach can trigger immune responses of the host but rarely leads to bacterial clearance in the absence of antibiotics, allowing it to survive in the stomach for decades. Its survival hinges on evading and manipulating the host immune system by altering surface molecules of the bacteria themselves to avoid detection by innate receptors like Toll-like receptors (TLRs) and RIG-I-like receptors (RLRs). The immune response against *H. pylori* infection starts with pattern recognition receptors (PRRs) on gastric epithelial and immune cells recognizing pathogen-associated molecular patterns (PAMPs), which leads to an adaptive response produced by the body (Larussa et al., 2015). *H. pylori* evades the natural immune response through several mechanisms, including evading recognition by TLRs and RLRs and activating inflammatory vesicle complexes (Karkhah et al., 2019; Salama et al., 2013). TLRs are usually located either on the plasma membrane surface or inside endosomes, where they bind to a range of PAMPs (Salama et al., 2013). The immune response to *H. pylori* begins by recognizing highly conserved PAMPs by PRRs on epithelial and intrinsic immune cells, after which an adaptive immune response is initiated (Karkhah et al., 2019).

**Figure 4:**
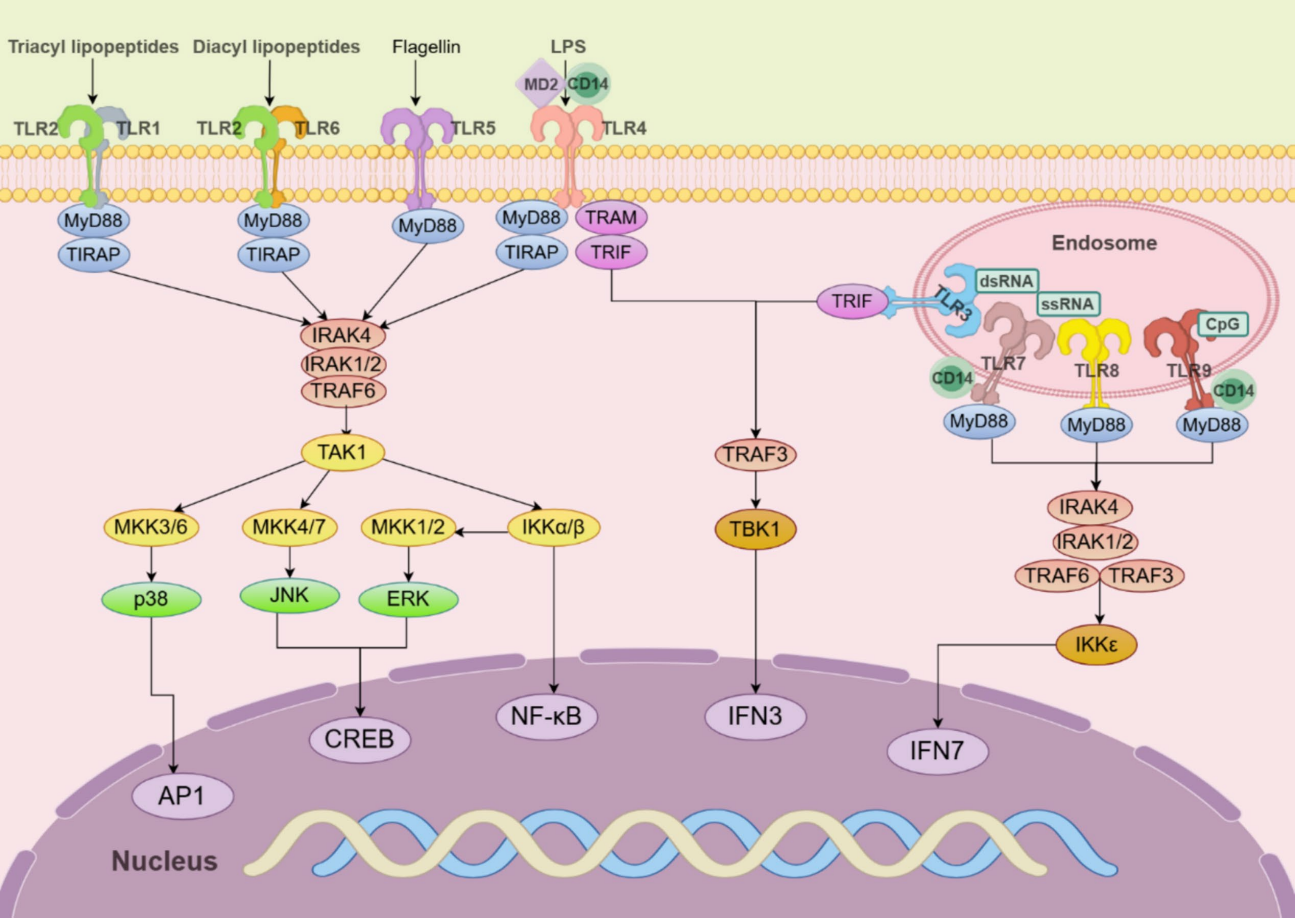
TLR signaling pathways in innate immune cells. TLR5, TLR4, and the heterodimers of TLR2/TLR1 or TLR2/TLR6 prefer to recognize the membrane components of pathogens at the cell surface, whereas TLR3, TLR7/TLR8, and TLR9 localize to the endosomes, where they recognize nucleic acids from both the host and foreign microorganisms. TLR4 localizes at the plasma membrane but is endocytosed into endosomes upon activation. MyD88 recruits downstream signaling molecules to form myddosome, which is based on MyD88 and contains IRAK4, IRAK1/2, and TRAF6.

Humans have 10 TLRs (TLR1–TLR10), while mice have 12 (TLR1–TLR9 and TLR11–TLR13). Some TLRs are distributed on the surface of cells (TLR1, TLR2, TLR4, TLR5, and TLR6), while others exist inside the cell, such as in the endoplasmic reticulum or endosomes (TLR3, TLR7, TLR8, and TLR9). Surface TLRs detect membrane components like lipids and proteins from microorganisms, while intracellular TLRs recognize nucleic acids from pathogens or the host. TLRs are found on all innate immune cells and also on adaptive immune cells of the host (Figure 4).

### 4.1 TLR2

TLR2 detects bacterial LPS, lipoproteins, and PGs, while *H. pylori* neutrophil-activating proteins stimulate a Th1 immune response via TLR2. For this function, TLR2 must bind to TLR1 and TLR6 to form a heterodimeric receptor complex on the cell surface (Chen et al., 2024). This activation triggers transcription factors like NF-κB, which promotes pro-inflammatory cytokines such as IL-1β, IL-2, IL-6, IL-8, and IL-12 (Meliț et al., 2019). Upon ligand binding, myeloid differentiation primary response protein 88 (MyD88) recruits members of the IL-1 receptor-associated kinase (IRAK) family (IRAK4, IRAK1, IRAK2) into the Myddosome complex. In some TLRs, coreceptors, including cluster of differentiation 14 (CD14) and myeloid differentiation factor 2 (MD2), facilitate ligand binding (Lim and Staudt, 2013). The Myddosome formation begins with activating IRAK4 through its N-terminal death structural domain, which then promotes sequential activation of IRAK1 and IRAK2. Activated IRAK1 interacts with tumor necrosis factor receptor-associated factor 6 (TRAF6), leading to TRAF6 activation. TRAF6 then recruits and activates the phosphorylation of transforming growth factor β-activated protein kinase 1 (TAK1) (Varga and Peek, 2017).

Activated TAK1 further phosphorylates and activates the classical IκB kinase (IKK) complex, ultimately leading to NF-κB activation. Second, activation of TAK1 leads to the activation of MAPKs, including MAPK kinases (MKK) 4/7 and MKK3/6, which further activate Jun N-terminal kinases and p38. Downstream TAK1 or IKK can be activated by the polyubiquitination of K63-linked TRAF6 and IRAK1. IKKβ activation leads to the activation of MKK1 and MKK2 and further activation of extracellular signal-regulated protein kinase (ERK) 1/2. These MAPKs are responsible for activating several transcription factors cyclic adenosine monophosphate-responsive element-binding protein (CREB) and activator protein 1 (AP-1) (Duan et al., 2022).

### 4.2 TLR4

TLR4, a member of the TLR family, plays a pivotal role in mediating the stomach inflammatory response induced by LPS derived from *H. pylori*. LPS is recognized by the dimeric complex TLR4/MD2, wherein TLR4 is associated with MD2, and MD-2 is essential for ligand binding and dimerization of TLR4. This recognition event triggers the activation of the NF-κB signaling pathway, which subsequently activates the IL-8 pathway, promoting the release of pro-inflammatory cytokines. Certain TLRs require coreceptors, such as CD14 and MD2, to effectively and stably bind their ligands. To evade detection by TLR4, *H. pylori* modifies the lipid A core of its LPS, altering its expression of ligands for the TLR4/MD2 immune complex and escaping potential immune clearance after TLR4 recognition (Meliț et al., 2019).

### 4.3 TLR5

TLR5 was identified as a receptor that recognizes bacterial flagellin, a protein component of polymerized flagellar filaments in certain Gram-positive and Gram-negative bacteria. *H. pylori* contains 5–7 flagella/cell, provides motility, and consists of two subunits: major flagellin and minor flagellin. Flagellin is a natural ligand of TLR5, specifically the highly conserved D1 domain at its N-terminus. Bacteria sometimes evade TLR5 sensing and recognition by down-regulating flagellin expression or mutating flagellin molecules. Mutations in the conserved structural domain of the *H. pylori* flagellin major subunit prevent TLR5 from recognizing it (Chen et al., 2024; Meliț et al., 2019). The mutation occurs between amino acids 89 and 96 in the D0-D1 structural domain and blocks *H. pylori* flagellum activation of TLR5 (Varga and Peek, 2017).

### 4.4 TLR7, TLR8, and TLR9

TLR9, an endosomal receptor, detects microorganisms and damaged cells via their glycocalyx, triggering an immune response (Meliț et al., 2019; Varga and Peek, 2017). *In vivo*, recognition of *H. pylori* by TLR9 can lead to a pro-inflammatory response, and conversely, TLR9 can promote anti-inflammatory signaling, thus acting as an inhibitor of *H. pylori*-induced gastritis and establishing persistent infection. Infection can lead to the release of DNA either actively or through degradation caused by host cells damaged by invading microbes. The binding of CpG DNA to mature cleaved TLR9 in endosomes induces conformational changes that lead to interactions between the homodimerized cytoplasmic Toll/IL-1 receptor structural domains (Varga and Peek, 2017). TLR7 recognizes resident microbiota, enhancing immunity, and along with TLR8, it detects *H. pylori* RNA, promoting gastrointestinal inflammation through MyD88. In TLR7, TLR8, and TLR9 signaling in plasmacytoid dendritic cells (pDCs), MyD88 also activates NF-κB signaling and interacts with the interferon regulatory factor IRF-7 to induce pro-inflammatory cytokine or type-I interferon (IFN-α and IFN-β) responses. A high level of IRF7 expression exists in pDCs, which binds to the Myddosome containing IRAK4, TRAF6, TRAF3, IRAK1, and IKK (Duan et al., 2022).

## 5 Antibiotic resistance

With the widespread use of medications, coupled with the specific adaptations of *H. pylori*, there is an increasing global report of antibiotic resistance in *H. pylori* and a decreasing eradication rate. Therefore, new antimicrobial drugs and therapeutic strategies are required to address this global problem.

### 5.1 Resistance to amoxicillin

**Figure 5:**
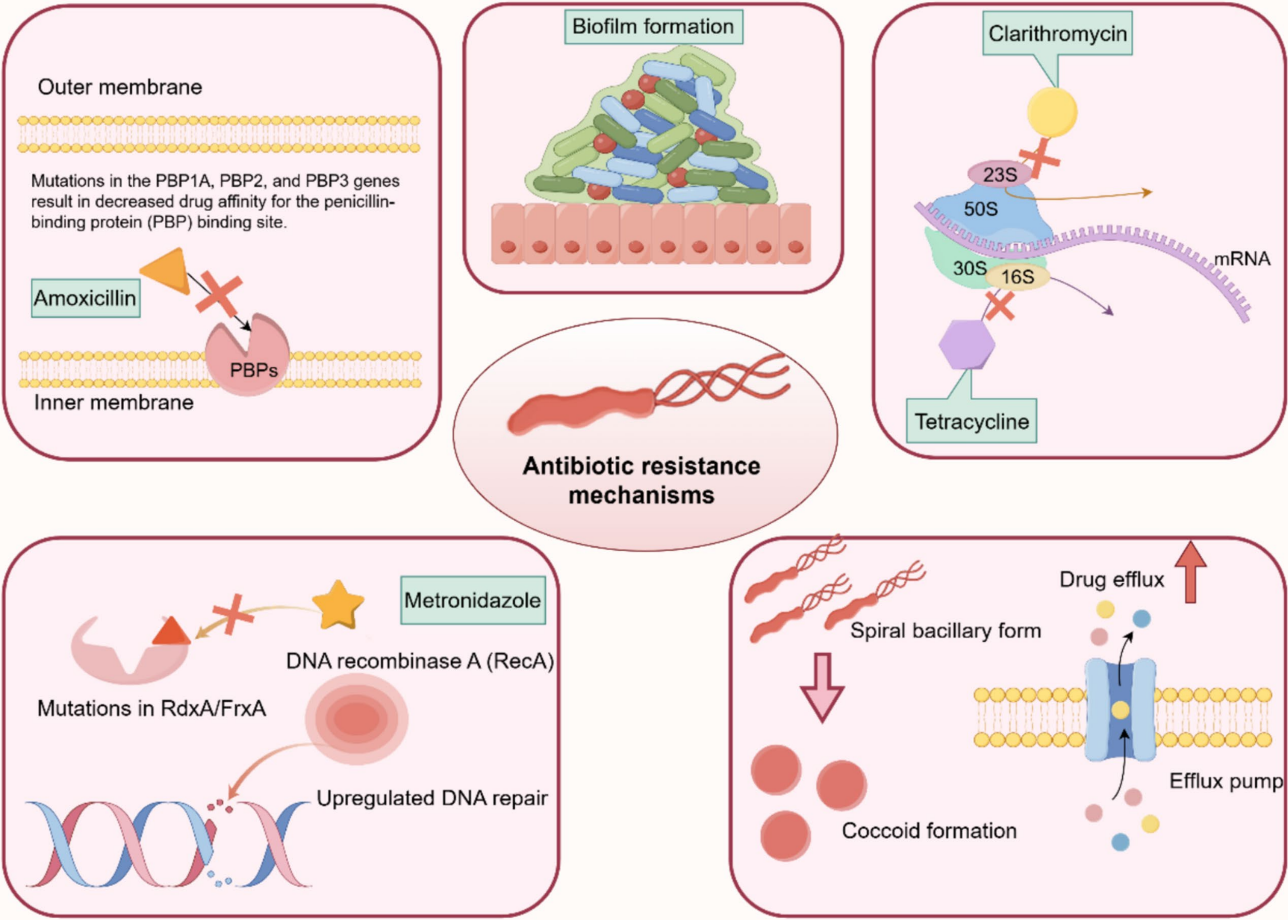
Molecular mechanism of drug resistance in *H. pylori*. The drug resistance mechanisms of *H. pylori* are mainly due to the following factors: (1) cumulative genetic mutations conferring resistance to each antibiotic, such as PBP genes, 23S rRNA, FrxA, and RdxA. (2) Upregulation of bacterial efflux pumps. (3) Downregulation of bacterial drug uptake pumps. (4) Biofilm or coccoid formation.

Amoxicillin (AMO) binds to penicillin-binding proteins (PBPs), impedes the synthesis of cell wall mucins, and interferes with the activity of transketolase enzymes required for cross-linking of nascent PG molecules, thereby inducing expansion and lysis of *H. pylori* cells (Figure 5) (Lin et al., 2023; Tshibangu-Kabamba and Yamaoka, 2021). *H. pylori* develops AMO resistance by several main mechanisms: mutations in the PBP gene, β-lactamase production, reduced drug penetration to the target site/membrane pore proteins, and mutations in the gene encoding the efflux pump (Lin et al., 2023). Mutations in PBP genes are the main mechanism of AMO resistance.

Moreover, mutations in pore proteins and efflux pumps encoding genes, including *hofH*, *hefC*, and *hopC*, which may alter membrane permeability to AMO in association with small-molecule solute diffusion. HefA, HefC, and HofH are associated with the efflux pump system, which mediates the increased efflux of antibiotics, and alterations in these proteins also lead to a decrease in cumulative drug concentrations in *H. pylori*, which can lead to AMO resistance. Some outer membrane proteins (HopB and HopC) are associated with small-molecule solute diffusion. Mutations in the genes encoding these proteins result in decreased membrane permeability, leading to a reduction in the concentration of drugs in *H. pylori*-infected cells (Lin et al., 2023). The production of β-lactamases is also an important factor in the development of AMO resistance and is mainly associated with the β-lactam antibiotic resistance enzyme TEM-1 (Lin et al., 2023; Ng et al., 2023; Tshibangu-Kabamba and Yamaoka, 2021).

### 5.2 Resistance to clarithromycin

Clarithromycin (CLA) is the most commonly used macrolide. Macrolides exert their bacteriostatic effect by reversibly binding to the peptidyl transferase ring of structural domain V of the 23S ribosomal RNA (rRNA) molecule in the bacterial ribosomal subunit 50S, interfering with protein synthesis. *H. pylori*’s resistance to CLA is most commonly due to point mutations in the 23S rRNA V region, particularly A2143G, A2142G, and A2142C. Point mutations in the 23S rRNA gene prevent CLA from binding to its 23S ribosomal subunit (Hasanuzzaman et al., 2024; Lin et al., 2023; Ng et al., 2023).

Another related mechanism of CLA resistance is attributed to the efflux pump system, where mutations in outer membrane proteins lead to decreased membrane permeability and increased efflux of antibiotics mediated by the efflux pump system, both resulting in decreased intracellular drug concentrations (Gong et al., 2024; Hasanuzzaman et al., 2024; Lin et al., 2023).

### 5.3 Resistance to tetracycline

The *H. pylori* resistance to tetracycline (TET) is primarily caused by mutations at positions 926–928 of 16S rRNA (Ng et al., 2023). In the bacterial cytoplasm, TET binds to the bacterial ribosome and interacts with the highly conserved 16S rRNA target in the 30S ribosome subunit, preventing translation and protein synthesis. Point mutations in 16S rRNA block TET binding to the main binding site of 16S ribosomes, which can lead to reduced drug affinity owing to their location at the main binding site (Hasanuzzaman et al., 2024). However, reports suggest that TET resistance manifests in the absence of mutated 16S rRNA genes, and the accumulation of the drug in the cytoplasm is reduced, implying the potential involvement of drug uptake restriction or efflux (Tshibangu-Kabamba and Yamaoka, 2021).

### 5.4 Resistance to metronidazole

Metronidazole (MET), a nitroimidazole antibiotic, is effective against *H. pylori* infection and remains active in gastric juice despite the low pH (Tshibangu-Kabamba and Yamaoka, 2021). MET activation produces nitro-anion free radicals that exhibit cytotoxic properties by directly damaging the subcellular structures and DNA of *H. pylori*. On the other hand, it can inhibit the proton dynamics of parietal cells and reduce ATP production in those cells (Ng et al., 2023). The mechanisms of MET resistance mainly include reduced drug uptake, enhanced DNA repair efficiency, counteraction of the damaging effects of MET metabolites, and overactivity of the oxygen clearance system (Gong et al., 2024; Tshibangu-Kabamba and Yamaoka, 2021).

### 5.5 Other possible mechanisms

Antibiotic resistance may not always be the cause of *H. pylori* eradication failure. An explanation for this phenomenon is the existence of *H. pylori* coccoid organisms that are induced by proton pump inhibitor (PPI). *H. pylori* appears to change its morphology to survive various adverse environmental conditions, including antibiotics, PPIs, and increased oxygen levels (Ierardi et al., 2020). The PG layer of the bacterial cell membrane plays an important role in maintaining bacterial morphology. Peptide modification of the PG layer occurs during the conversion of *H. pylori* to coccoid forms (Brkić et al., 2024). It has been demonstrated that the quantity of coccoid forms is linked to the levels of PPIs and antibiotics. When omeprazole is removed from the culture medium, the viability of *H. pylori* improves, and the spiral form changes (Krzyżek and Grande, 2020).

Moreover, antibiotic resistance is associated with the formation of biofilms. These adhesive aggregates of *H. pylori*, surrounded by layers of extracellular polymers, create a stable protective surface attachment. Mutations in genes related to the SpoT enzyme, α-(1,3)-fucosyltransferase, and other proteins enhance biofilm formation and antibiotic resistance in *H. pylori* (Hasanuzzaman et al., 2024; Hathroubi et al., 2018; Lin et al., 2023).

Many pathogenic bacteria exhibit antibiotic resistance through efflux pumps. Efflux pumps regulate the internal environment by extruding toxic substances, quorum-sensing molecules (autoinducers), biofilm-forming molecules, and virulence factors (Gaurav et al., 2023). The biological function of bacterial efflux pumps is linked to the efflux of antibiotics and the formation of biofilms (Gaurav et al., 2023; Lin et al., 2023).

## 6 Treatment methods for *Helicobacter pylori* infection

### 6.1 Standard triple therapy

Standard triple therapy (STT) is one of the most commonly used treatment methods for *H. pylori* eradication and consists of a PPI and two antibiotics. Commonly used PPIs include omeprazole, lansoprazole, pantoprazole, rabeprazole, and esomeprazole (Liang et al., 2022). Commonly used antibiotics include AMO, CLA, levofloxacin, rifabutin, and MET. However, with increasing antibiotic resistance, these drug-based triple regimens have been unable to eradicate *H. pylori* at rates of more than 80%, even if the regimen is prolonged to 10 or 14 days (d) (Supplementary Table S1). STT should generally be continued for 10–14 days until *H. pylori* is completely eradicated. Many bacteria, including *H. pylori*, have developed resistance to antibiotics because of the widespread indiscriminate use of antibiotics (Sun et al., 2023; Suzuki et al., 2022).

Drug susceptibility testing is the best way to optimize and minimize antibiotic use in the treatment of *H. pylori*, significantly improving eradication rates. However, due to the invasive nature of endoscopy, it is not a routine practice and may be harmful, time-consuming, and costly. *H. pylori* eradication therapy may also disrupt gut microbiota, as antibiotics and PPIs affect gut microbiota through their antimicrobial action and reduction of gastric acidity (Suzuki et al., 2022). In fact, a study showed that patients undergoing triple therapy, which typically includes two antibiotics and a PPI, experienced a significant drop in gut microbiota diversity during the first eight weeks, with recovery occurring afterward (Liou et al., 2019).

### 6.2 Bismuth quadruple therapy

Typically, PPI, a bismuth agent, and two antibiotics comprise a bismuth quadruple regimen. The classic bismuth quadruple therapy regimen, consisting of a PPI, bismuth, TET, and MET, was initiated in 1995 and established before the CLA triple therapy regimen (Liang et al., 2022; Sun et al., 2023). With the emergence of *H. pylori* resistance to CLA, triple therapy, including CLA, has become less effective, making bismuth quadruple therapy the preferred choice. There is no doubt that bismuth quadruple therapy can be a powerful tool in the fight against CLA and MET resistance. As this regimen does not contain CLA, MET resistance is likely to be overcome by the synergistic action of MET and bismuth (Zagari et al., 2023). Bismuth is a mucosal protectant against *H. pylori* by inhibiting some of its enzymes, including urease, fumonisinase, ethanol dehydrogenase, and phospholipase. Despite its resistance to other antibiotics, bismuth remains a reliable agent in regions where retreatment is necessary, and antibiotic resistance is common. Bismuth increases the eradication rate of drug-resistant *H. pylori* strains by 30–40% (Liang et al., 2022).

### 6.3 Concomitant, sequential, and dual therapies

Using four drugs (a PPI and three antibiotics) together during concomitant therapy is often referred to as “non-bismuth quadruple therapy.” A recent consensus conference organized by the Hellenic Society of Gastroenterology recommended that a non-bismuth quadruple regimen consisting of a PPI, AMO, CLA, and MET for at least 10 days should be the ideal first-line treatment in Greece, as it leads to higher *H. pylori* eradication rates (Viazis et al., 2022). Dual therapy (DT) comprises an acid suppressant and an AMO. This methodology, which combines PPIs with AMO (Hu, 2022). Numerous studies have proven the significance of high-frequency AMO utilization in high-dose dual therapy (HDDT), indicating a positive association between high-dose PPIs and *H. pylori* eradication rates in DT. In HDDT, acid suppressors are administered simultaneously with at least 3,000 mg of AMO (Duan et al., 2023). HDDT is a promising treatment that can reduce unnecessary antibiotic use. The 2022 Chinese clinical practice guidelines for *H. pylori* eradication therapy recommend HDDT (Zhou et al., 2022). One limitation of DT is that it can only be used by people without penicillin allergies. Another constraint is that DT remains in its infancy and has not been adopted extensively (Duan et al., 2023).

The sequential treatment challenges the traditional STT. Sequential therapy involves a unique strategy in which PPIs and AMO are administered twice daily for the first 5 days, followed by a combination of PPIs, CLA, and nitroimidazoles twice daily for the next 5 days. Since 2007, STT eradication rates have been below 80%, which is considered a disappointing level for the resolution of antimicrobial infections, and sequential therapy has been recommended as an alternative to STT for *H. pylori* infection (Nyssen et al., 2024; Yang et al., 2014).

### 6.4 Probiotics

**Figure 6:**
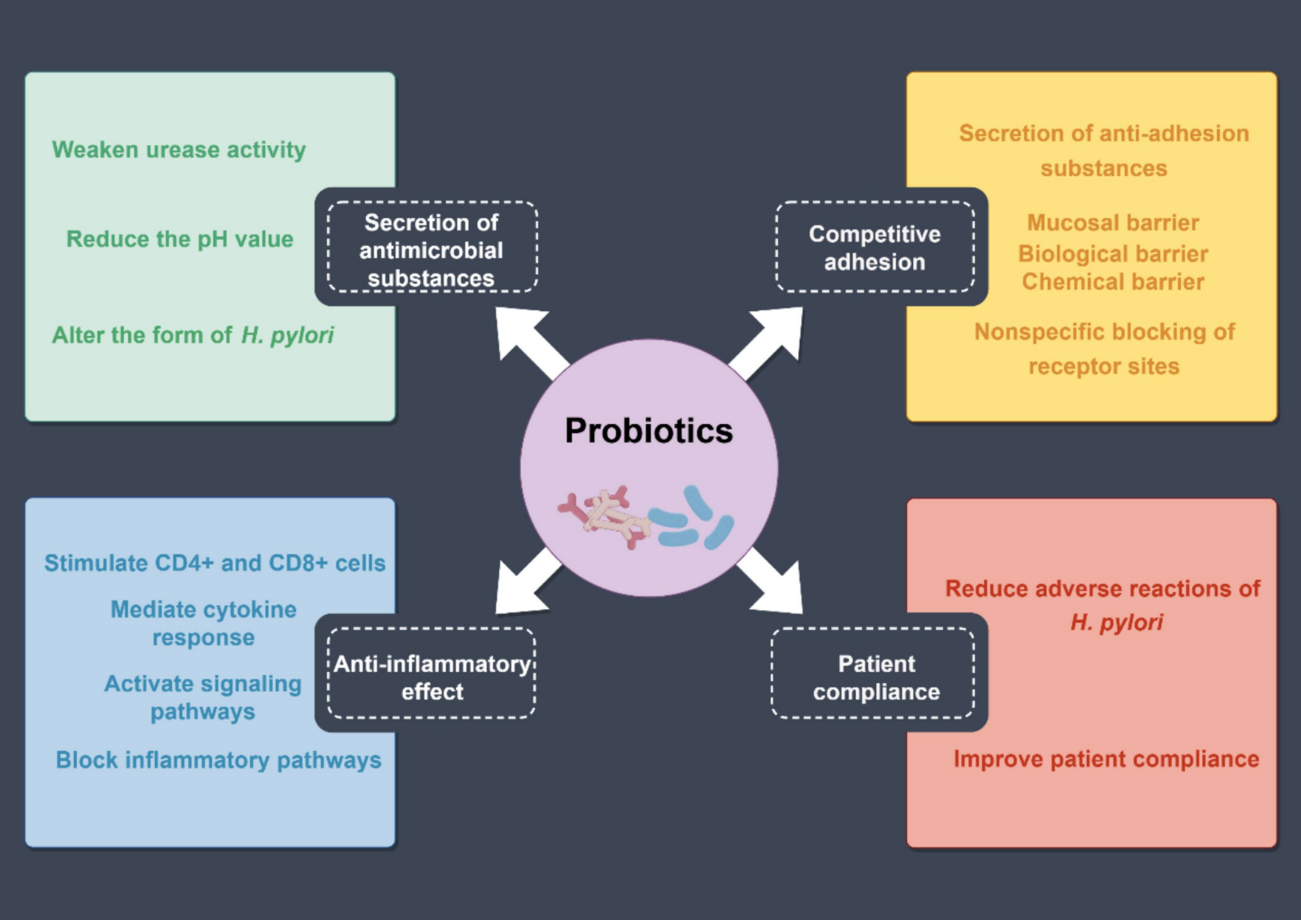
Mechanism of probiotics to increase the eradication rate of *H. pylori*. The effects of probiotics can be summarized into four aspects: the secretion of antimicrobial substances, competitive adhesion, patient compliance, and anti-inflammatory effects.

Probiotics are a class of microorganisms beneficial to human health (Liang et al., 2022). Common probiotics include *Lactobacillus*, *Bifidobacterium*, *Streptococcus*, and *Yeast* (Nabavi-Rad et al., 2022). Studies have revealed that probiotic preparations combined with antibiotics can effectively alleviate *H. pylori* infection patients’ clinical symptoms, improve the *H. pylori* eradication effect, and reduce the incidence of adverse drug reactions (Figure 6).

The theoretical basis for the inhibition of *H. pylori* by probiotics mainly includes the following factors: (1) secretion of antimicrobial substances: probiotics produce antimicrobial substances, including hydrogen peroxide, bacteriocins, and short-chain fatty acids, which reduce intragastric pH values by attenuating urease activity and altering *H. pylori* morphology, thereby inhibiting the colonization (Liang et al., 2022; Xu et al., 2022). (2) Competitive adhesion: probiotics can secrete anti-adhesion substances, and produce adhesion factors. They competitively inhibit *H. pylori* by binding to the binding sites of gastric mucosal epithelial cells, thereby preventing *H. pylori* adhesion. Moreover, probiotics and their active substances adhere closely to the gastric mucosal surface to form a biochemical barrier. (3) Anti-inflammatory effect: probiotics stimulate the activation and proliferation of CD4^+^ and CD8^+^ cells in the lamina propria of the mucosa, generate cellular immune response, increase secretory immunoglobulin A production, and promote the proliferation of epithelial cells to accelerate the repair and regeneration of the mucosa. Furthermore, probiotics can increase the expression of cytokine signaling inhibitors to block *H. pylori* signaling (Nabavi-Rad et al., 2022; Xu et al., 2022). (4) Patient compliance: probiotic preparations can reduce antibiotic-induced taste disorders, diarrhea, nausea, abdominal pain, constipation, and other adverse reactions and significantly improve patients’ *H. pylori* eradication treatment compliance, thereby improving the *H. pylori* eradication rate (Chen et al., 2018; Xu et al., 2022).

### 6.5 Traditional medicine therapy

Traditional Chinese medicine (TCM) effectively treats gastric diseases, protects spleen and stomach functions, and causes few adverse reactions. In clinical practice, physicians treating patients with drug-resistant *H. pylori* infection may contemplate the addition of herbal medicines after consulting with patients (Zhou et al., 2022). In TCM, disease is caused by imbalances of positive and evil Qi. *H. pylori* infection symptoms are classified under conditions like “stomach pain” and “acid reflux.” *H. pylori* is seen as a “damp-heat pathogen” with “toxicity” (Zhao et al., 2021).

TCM uses active ingredients like alkaloids, polyphenols, flavonoids, and terpenoids for gastritis treatment. Alkaloids, in particular, offer gastric protection through anti-inflammatory, antioxidant, anti-apoptotic, antimicrobial, and mucosal protection activities. Berberine and palmatine are alkaloids from herbs like *Coptis chinensis* (Guo et al., 2024). Quercetin, kaempferol, and baicalin exhibit antioxidant, anti-inflammatory, and immunomodulatory activities. Ginsenoside Rg1 effectively inhibits atrophy and inflammation. *Astragalus* and *licorice* polysaccharides are commonly used as antioxidant and anti-apoptotic agents (Zhao et al., 2021). The primary mechanisms of action of herbal medicines include inhibiting the replication and transcription of *H. pylori*, reducing urease expression, destroying the bacterial structure, down-regulating virulence factors expression, and inhibiting signaling protein activation (Li et al., 2021; Sun et al., 2023).

African traditional medicine remains one of the oldest cultural practices, and many natural products have been recommended for the treatment of *H. pylori*-related gastric ulcers. Among them, the calyx extract of *Hibiscus sabdariffa* L. and the root extract of *Terminalia macroptera* Guill. & Perr. were found to have noteworthy antimicrobial activity. Also, the South African honey variants and Pure Honey were found to possess excellent antibacterial effects (Dinat et al., 2023). Moreover, according to Iranian traditional medicine, extracts from *Sambucus ebulus* and *Rheum ribes* are highly effective at inhibiting urease, which helps the stomach improve its acidic environment and enables the immune system to naturally eliminate *H. pylori* (Nabati et al., 2012).

### 6.6 Nanotechnology

**Figure 7:**
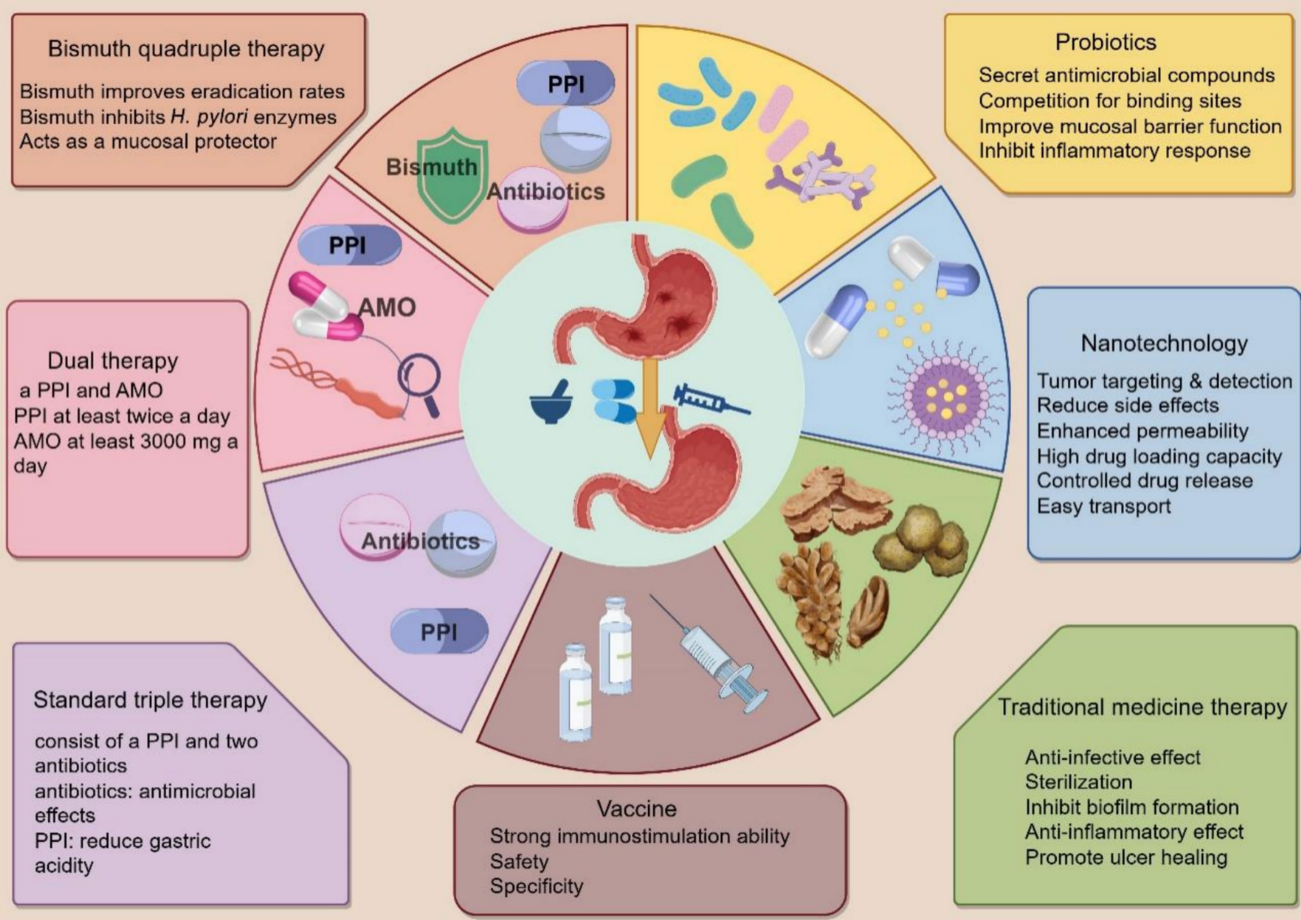
Treatment options for *H. pylori*. Triple therapy, bismuth quadruple therapy, concomitant therapy, and dual therapy are traditional experiential therapies for *H. pylori*. Probiotics, nanotechnology, and traditional herbal medicines are essential adjuvant therapies. Specific vaccines can prevent *H. pylori* infection.

Oral medication is a common treatment for *H. pylori*; however, certain antibiotics deteriorate in the gastric environment and become ineffective. Nanomaterials, due to their small size and high permeability, protect and deliver drugs effectively, often in capsule form (Sun et al., 2023). They allow for the loading of multiple drugs for synergistic therapy and offer benefits like active targeting, tumor detection, reduced side effects, enhanced retention, high drug capacity, controlled release, and easy tissue penetration. Available nanomaterials include graphene, gold nanoparticles, metal-based nanoparticles (Yin et al., 2023), silicon dioxide nanoparticles, polymer nanoparticles, and liposomes (Rai et al., 2021). However, more research is needed on their biodegradability, biocompatibility, and potential toxicity. Moreover, metal nanoparticles, quantum dots, and other nanomaterials are not biodegradable, resulting in their prolonged presence in the body after administration and enhanced cytotoxicity (Figure 7).

### 6.7 *Helicobacter pylori* vaccine

Research into an *H. pylori* vaccine has become a notable and active field within antibiotic replacement therapy. Two main vaccine approaches are a whole-cell bacterium and a recombinant preparation that includes protective antigens and immune adjuvants. Various immune adjuvants, such as BabA, SabA, OipA, CagA, and VacA, enhance vaccine efficacy against *H. pylori*. Targeting OMPs is a promising treatment strategy, with HopB and HopC as potential vaccine targets. Proteins HopV, HopW, HopX, and HopY are stable immunogens. Disrupting BabA and SabA interactions with the mucosa can improve the therapeutic effect of antibiotics on *H. pylori*. Porcine milk and rhamnogalacturonans show potential in inhibiting SabA and BabA, respectively. Additionally, OipA is a promising oral vaccine candidate, with IgA against it improving infection outcomes in mice. OMVs can act as vaccine adjuvants, inducing immune responses without causing disease due to their inability to replicate. Progress in therapies targeting CagA includes focusing on the CagA secretion system using inhibitors of ATPase CagA (Sedarat and Taylor-Robinson, 2024; Zhang et al., 2024). Although there has been some progress in researching vaccines targeting the virulence factors of *H. pylori*, a long way remains before safe, non-toxic, and effective vaccines can be clinically promoted. Further exploration and experimentation by research teams worldwide are still needed.

## 7 Conclusion

This study provides a comprehensive analysis of the pathogenic mechanism of *H. pylori*, examining five aspects: colonization, virulence factors, immunity, drug resistance, and treatment. Since the discovery of *H. pylori*, our understanding of the bacterium has progressed significantly; however, additional research is required to explain its pathogenesis. Further elucidation is required regarding the interaction between Cag T4SS and host cells, necessitating a clearer understanding of the mechanism by which Cag T4SS-positive *H. pylori* strains promote the onset of digestive diseases. This article presents certain pathogenic mechanisms of *H. pylori*, while others may remain unexplored, offering a partial response to this inquiry. Because *H. pylori* comprise approximately 1,600 genes, it remains possible to identify additional important disease-causing genes. *H. pylori*-related diseases are also closely related to host factors, including diet and environmental conditions. In addition, the overview of virulence factors involved in the pathogenesis is much broader than what is mentioned in the article. As *H. pylori* exhibits increasing resistance to antibacterial drugs, it is crucial to monitor the progress of its resistance mechanisms, investigate novel and effective alternative medications and therapies, and research and develop specific vaccines.

## Glossary

CagA: Cytotoxin-associated antigen A
Cag PAI: Cytotoxin-associated gene pathogenicity island
VacA: Vacuolating cytotoxin
OMPs: Outer membrane proteins
PG: Peptidoglycan
EPIYA: Glu-Pro-Ile-Tyr-Ala
PPI: Proton pump inhibitor
SabA: Salic acid-binding adhesin
BabA: Blood group antigen-binding adhesin
OMCC: Outer membrane core complex
IMC: Inner membrane complex
PAMPs: Pathogen-associated molecular patterns
IL: Interleukin
OipA: Outer membrane inflammatory protein
Leb: Lewis b
OMVs: Outer membrane vesicles
ICAM-1: Intercellular cell adhesion molecule-1
ECP: Eosinophil cationic protein
NF-κB: Nuclear factor-κB
ATF2: Activated transcription factor 2
MAPK: Mitogen-activated protein kinases
ATP: Adenosine-triphosphate
AJs: Adhesion junctions
TJs: Tight junction
PAR1b: Polar-regulated kinase 1b
T4SS: Type IV secretion system
TRPML1: Transient receptor potential membrane channel mucolipin 1
sLex: Sialylated Lewis x
PRR: Pattern recognition receptor
TLRs: Toll-like receptors
RLRs: RIG-like receptors
TRAF6: Tumor necrosis factor receptor-associated factor 6
IRAK: IL-1 receptor-associated kinases
LPS: Lipopolysaccharide
TNF-α: Tumor necrosis factor-α
HDDT: High-dose dual therapy
DT: Dual therapy
STT: Standard triple therapy
MET: Metronidazole
TET: Tetracycline
rRNA: Ribosomal RNA
CLA: Clarithromycin
PBPs: Penicillin-binding proteins
AMO: Amoxicillin
MD2: Myeloid differentiation factor 2
AMP: Ampicillin
H^+^/K^+^-ATPase: Hydrogen-potassium triphosphatase
ERK: Extracellular signal-regulated protein kinase
pDCs: Plasmacytoid dendritic cells
MyD88: Myeloid differentiation primary response protein 88
CD14: Cluster of differentiation 14
IKK: IκB kinase
TAK1: Transforming growth factor β-activated protein kinase 1
MKK: MAPK kinases
AP-1: Activator protein 1
CREB: Cyclic AMP-responsive element-binding protein
TCM: Traditional Chinese medicine
