# Supplementary material for: Helicobacter pylori infection process: from the molecular world to clinical treatment
Source: Front Microbiol. 2025 Feb 27;16:1541140. doi: 10.3389/fmicb.2025.1541140 (PMC11903457; doi:10.3389/fmicb.2025.1541140)
Supplement: Supplementary file 1 [file Table_1.docx]

**Supplementary Table 1.** Currently used first-line *H. pylori* treatment regimens.

| Therapy | Components | d | Eradication rates | | Comments & recommendations |
| --- | --- | --- | --- | --- | --- |
|  |  |  | **ITT** | **PP** |  |
| Bismuth quadruple therapy | Lansoprazole (30 mg bid) Bismuth (300 mg qid)  TET (500 mg qid)  MET (500 mg tid) | 14 | 74.3% (Kim et al., 2021) | 92.9% (Kim et al., 2021) | Recommended for first and second-line eradication treatment in people with *H. pylori* infection and patients with previous MA exposure or who are allergic to AMP. |
| Non-bismuth quadruple therapy | Esomeprazole (40 mg bid) AMO (1000 mg bid)  CLA (500 mg bid)  MET (500 mg bid) | 10 | 92.3% (Zeriouh et al., 2020) | 95.3% (Zeriouh et al., 2020) | Not recommended for patients with CLA resistance or AMP allergy. |
| Standard triple therapy | Lansoprazole (30 mg bid)  CLA (500 mg bid)  AMO (1000 mg bid) | 7 | 60% (Sezgin et al., 2019) | 57% (Sezgin et al., 2019) | Recommended for low CLA resistance (15%) and patients with no previous history of MA exposure. |
| Vonoprazan–AMO dual therapy | Vonoprazan (20 mg bid)  AMO (750 mg qid) | 14 | 94.6% (X. Wang et al., 2023) | 98.5% (X. Wang et al., 2023) | Efficacy in first-line eradication is not inferior to bismuth quadruple therapy and is not affected by CLA resistance, with fewer adverse effects. |
| Vonoprazan-based  triple Therapy | Vonoprazan (20 mg bid)  AMO (750 mg bid)  CLA (200 mg bid) | 7 | 81.5% (Okubo et al., 2020) | 90.8% (Okubo et al., 2020) | Effective and well-tolerated, irrespective of CLA resistance. Recommended as a first-line treatment regimen for *H. pylori* infection. |
| Tegoprazan-based  triple therapy | Tegoprazan (50 mg bid)  CLA (500 mg bid)  AMO (1000 mg bid) | 7 | 62.86% (Choi et al., 2022) | 69.3% (Choi et al., 2022) | As effective as standard triple therapy and is as safe as first-line *H. pylori* eradication therapy, but it does not overcome the CLA resistance of *H. pylori*. |
| Tegoprazan-based concomitant therapy | Tegoprazan (50 mg bid)  CLA (500 mg bid)  AMO (1000 mg bid)  MET (500 mg bid) | 10 | 90.5% (Kwon et al., 2023) | 96.2% (Kwon et al., 2023) | Beneficial for patients with dual MET/CLA resistant strains.  Recommended as an effective first-line therapy for overcoming antibiotic resistance. |
| Sequential therapy | Esomeprazole (40 mg bid)  AMO (1000 mg bid) for 7 d, followed by Esomeprazole (40 mg bid)  CLA (500 mg bid)  Tinidazole (500 mg bid) for last 7 d | 14 | 90.3% (Zullo et al., 2019) | 97% (Zullo et al., 2019) | Recommended for bacteria with single resistance to clarithromycin or MET. |
| High-dose esomeprazole and AMO dual therapy | Esomeprazole (40 mg bid)  AMO (1000 mg tid) | 14 | 64.6% (Zeriouh et al., 2020) | 67.7% (Zeriouh et al., 2020) | Recommended for initial and second eradication treatments. |

AMO, amoxicillin; CLA, clarithromycin; ITT, intention-to-treat; MET, metronidazole; TET, tetracycline; P-CAB, potassium-competitive acid blocker (vonoprazan); AMP, ampicillin; PP, per-protocol; MA, macrolide antibiotics; PPI, proton pump inhibitor; qd, once daily; bid, twice daily; qid, four times daily; tid, three times daily.
